# Signalling by co-operative higher-order assembly formation: linking evidence at molecular and cellular levels

**DOI:** 10.1042/BCJ20220094

**Published:** 2025-03-05

**Authors:** Bostjan Kobe, Jeffrey D. Nanson, Mikayla Hoad, Antje Blumenthal, Yann Gambin, Emma Sierecki, Katryn J. Stacey, Thomas Ve, Randal Halfmann

**Affiliations:** 1 School of Chemistry and Molecular Biosciences, The University of Queensland, Brisbane, QLD 4072, Australia; 2 Australian Infectious Diseases Research Centre, The University of Queensland, Brisbane, QLD 4072, Australia; 3 Institute for Molecular Bioscience, The University of Queensland, Brisbane, QLD 4072, Australia; 4 Gulbali Institute, Charles Sturt University, Wagga Wagga, NSW 2678, Australia; 5 Frazer Institute, Faculty of Medicine, The University of Queensland, Brisbane, QLD, 4102, Australia; 6 School of Biomedical Sciences, The University of New South Wales, Sydney, NSW 2052, Australia; 7 Institute for Biomedicine and Glycomics, Griffith University, Gold Coast, QLD 4215, Australia; 8 Stowers Institute for Medical Research, Kansas City, MO 64110, U.S.A.; 9 Department of Biochemistry and Molecular Biology, University of Kansas Medical Center, Kansas City, KS 66103, U.S.A.

**Keywords:** death fold domain, innate immunity, signalling by co-operative assembly formation (SCAF), signalosome, TIR domain, RHIM

## Abstract

The concept of higher-order assembly signalling or signalling by co-operative assembly formation (SCAF) was proposed based on the structures of signalling assemblies formed by proteins featuring domains from the death-fold family and the Toll/interleukin-1 receptor domain family. Because these domains form filamentous assemblies upon stimulation and activate downstream pathways through induced proximity, they were envisioned to sharpen response thresholds through the extreme co-operativity of higher-order assembly. Recent findings demonstrate that a central feature of the SCAF mechanism is the nucleation barrier that allows a switch-like, digital or ‘all-or-none’ response to minute stimuli. In agreement, this signalling mechanism features in cell-death and innate immunity activation pathways where a binary decision is required. Here, we broaden the concept of SCAF to encapsulate the essential kinetic properties of open-ended assembly in signalling, compare properties of filamentous assemblies and other co-operative assemblies such as biomolecular condensates, and review how this concept operates in cells.

## Introduction

The concept of signalling through higher-order protein assembly emerged from the structural biology of death-fold (DF) domains involved in cell-death and immune-cell activation pathways. The proteins were observed to form complexes such as the PIDDosome, the Fas:FADD complex and the Myddosome (see Table 1 for components and description of the abbreviations) [1,2,4,13]. The concept later extended to signalosomes formed by Toll/interleukin-1 receptor (TIR) domains [7,8] and receptor-interacting protein [RIP] homotypic interaction motifs (RHIMs) [11]. The term signalling by co-operative assembly formation (SCAF) was originally proposed to capture the functional logic of such assemblies through the biochemical property of co-operativity [14,15], which describes how interdependent subunits in an assembly can create ultrasensitive responses to stimuli. Recent findings reveal that some DF assemblies involve nucleation barriers that allow for the maintenance of components at a level that enables rapid assembly of a large signalosome, once triggered by a template seed [16,17]. Here, we broaden the SCAF concept to include this newly appreciated kinetic function of filamentous open-ended assemblies and discuss how co-operativity and nucleation barriers, together, provide the required sensitivity, speed and output level required for innate immunity and programmed cell-death signalling.

**Table 1:** Representative SCAF-related assemblies

	Components	Signalling pathway	References
**DF domain assemblies**			
PIDDosome	PIDD, p53-induced protein with a death domain	Apoptosis	[1]
Fas:FADD complex	FS-7 [foreskin] cell line-associated surface antigen, Fas-associated death domain protein	Apoptosis	[2]
DISC (death-inducing signalling complex)	DDs of death receptor and FADD, DEDs of FADD and caspase-8	Apoptosis	[3]
Myddosome	DDs of MyD88, IRAK2 and IRAK4	TLR and IL-1R (interleukin-1 receptor) pathways	[4]
CBM (CARD–BCL10–MALT1) signalosome	CARDs of CARD9/10/11/14, BCL10 and DD of MALT1 (mucosa-associated lymphoid tissue lymphoma translocation protein 1)	CLR, TCR and BCR pathways	[5]
Inflammasome	PYDs of AIM2, NLRs such as NLRP3 and ASC, and CARDs of ASC and caspase-1	Pyroptosis	[6]
**TIR domain assemblies**			
MAL, MyD88, TRIF and TRAM filaments	MAL, MyD88, TRIF and TRAM TIR domains	TLR pathways	[7–9]
SARM1 octamers	SARM1 TIR domains	Axon degeneration	[10]
**RHIM assemblies**			
Protein kinases RIPK1 (receptor-interacting serine/threonine-protein kinase 1) and RIPK3	RIP1 and RIP3 RHIMs	Necroptosis	[11,12]

Observations of SCAF predate the acronym itself. Signalling via polymerization was originally described as ‘higher-order assembly signalling’ [13]. The assemblies themselves were termed supramolecular organizing centres (SMOCs) [18], although the term signalosome has become more commonly used. Names for specific signalosomes have in some cases become widely adopted, for example, inflammasome [19], necrosome [20], Myddosome [4] and resistosome [21], among others. ‘SMOC’ emphasizes the roles of signalosomes in orchestrating downstream signalling. Recent findings suggest, however, that signalosome function and, hence, structure emerge more from thermodynamic and kinetic properties of homotypic assembly than on physical connectivity of different signalling components [17,22]. We consequently prefer the acronym ‘SCAF’, as it more closely aligns with the functional logic of these assemblies.

The structural basis of signalosome assembly has been the subject of several reviews (e.g. [13,18,23–26]). In the present study, we briefly present illustrative examples of the structural domains that employ this signalling mechanism and the pathways they feature in. We, then, describe key thermodynamic and kinetic principles of the mechanism and its implications for cellular signalling and compare it with ones involving other co-operative assemblies such as biomolecular condensates. We, then, focus on the evidence that this signalling mechanism operates in cells and briefly discuss the implications for pathology. We end by highlighting some open questions. Note that MyD88 features more frequently than other systems in the present study, in part because of the abundance of data available on this system, as well as because it contains two classes of SCAF domains in the same protein (a death domain [DD] and a TIR domain). As such, we also selected MyD88 as a case study for discussion regarding therapeutic development.

### SCAF signalosome architecture

**Figure 1:**
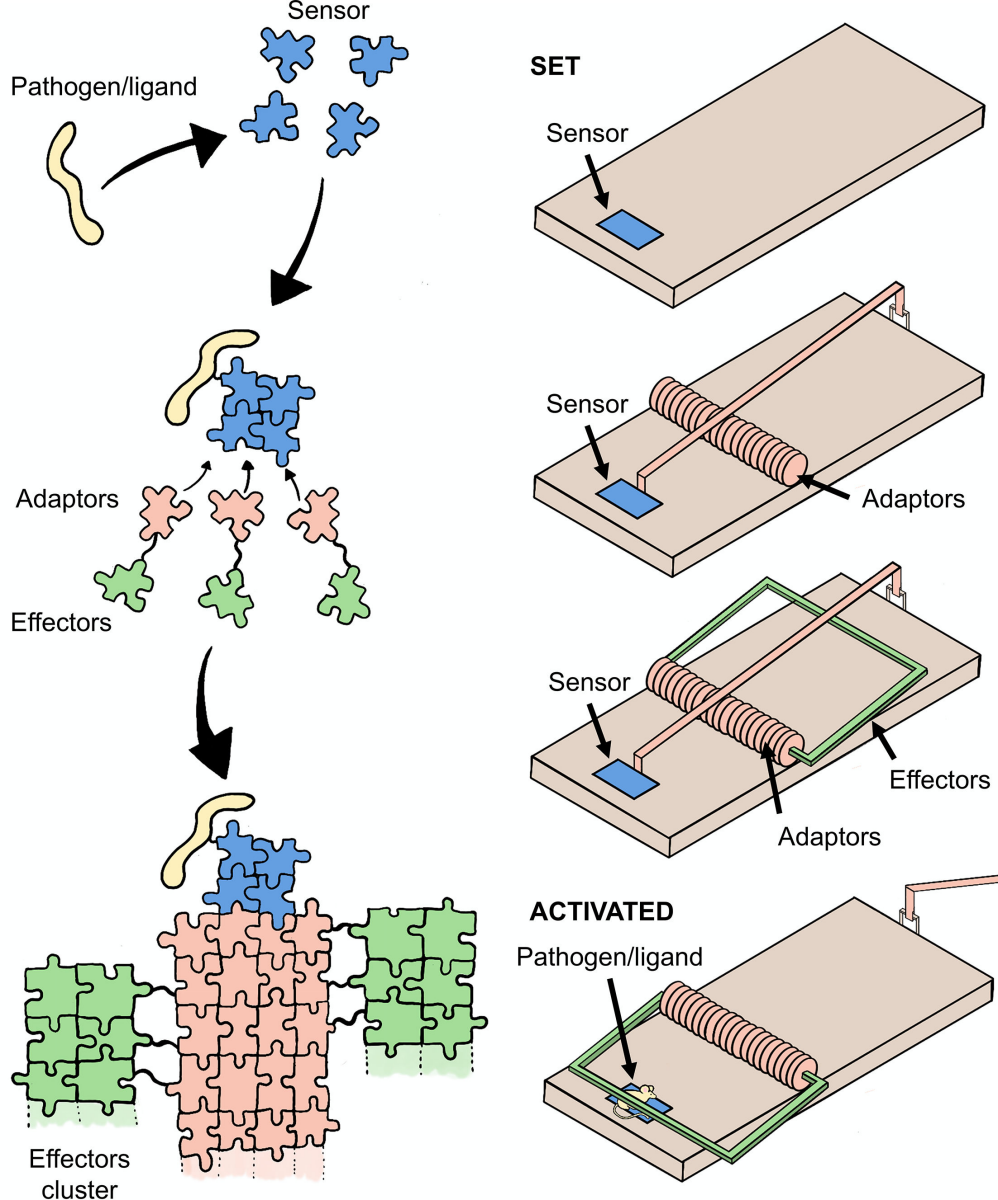
Schematic diagram of the SCAF mechanism and analogy to a mousetrap. Signalosomes typically contain three components: a sensor, an adaptor and an effector. The sensor recognizes signs of danger, such as components of pathogens, and relays that signal via the adaptor to an effector protein that carries out the cellular response. The oligomerization of the sensor provides the template for the assembly formation of adaptor proteins, which are present at supersaturating concentrations. This assembly in turn brings together effector proteins, resulting in their proximity-based activation and cellular response. In the mousetrap, the sensor protein represents the trigger; the adaptor represents the spring; and the effectors represent the hammer (shown in colours matching the schematic diagram). The spring is loaded through a supersaturating concentration of adaptor proteins. Upon the sensor sensing the pathogen (‘mouse’), the spring releases its stored energy and propels the hammer onto the mouse.

Signalosomes typically contain at least three components: a sensor, an adaptor and an effector (Figure 1). These components often comprise different proteins, but sometimes two or more components are linked together in the same protein. The sensor recognizes signs of danger, such as components of pathogens or mislocalized cellular molecules (pathogen- or damage-associated molecular patterns [PAMPs or DAMPs], respectively), or alternatively cellular aberrations induced by infection, and relays that signal via an intervening protein known as an adaptor, to an effector protein that carries out the cellular response. The classes of sensors in innate immunity include the Toll-like receptor (TLR), nucleotide-binding oligomerization domain-like receptor (NLR), RIG-I (retinoic acid-inducible gene 1)-like receptor (RLR), C-type lectin domain receptor and AIM (absent in melanoma) 2-like receptor. The antigen receptors of the acquired immune system, the B-cell receptor and T-cell receptor (TCR), also use SCAF in some downstream pathways. Effector proteins tend to be enzymes that undergo intermolecular auto-activation following their induced proximity on the surface of adaptor assemblies. They include auto-proteolytic caspases, self-activating kinases and TIR-domain NADases that bind and cleave the dinucleotide NAD^+^ (nicotinamide adenine dinucleotide) when self-associated [27–29]. The cellular consequences executed by effector activation include but are not limited to pro-inflammatory gene transcription, cytokine release and multiple forms of programmed cell death.

**Figure 2:**
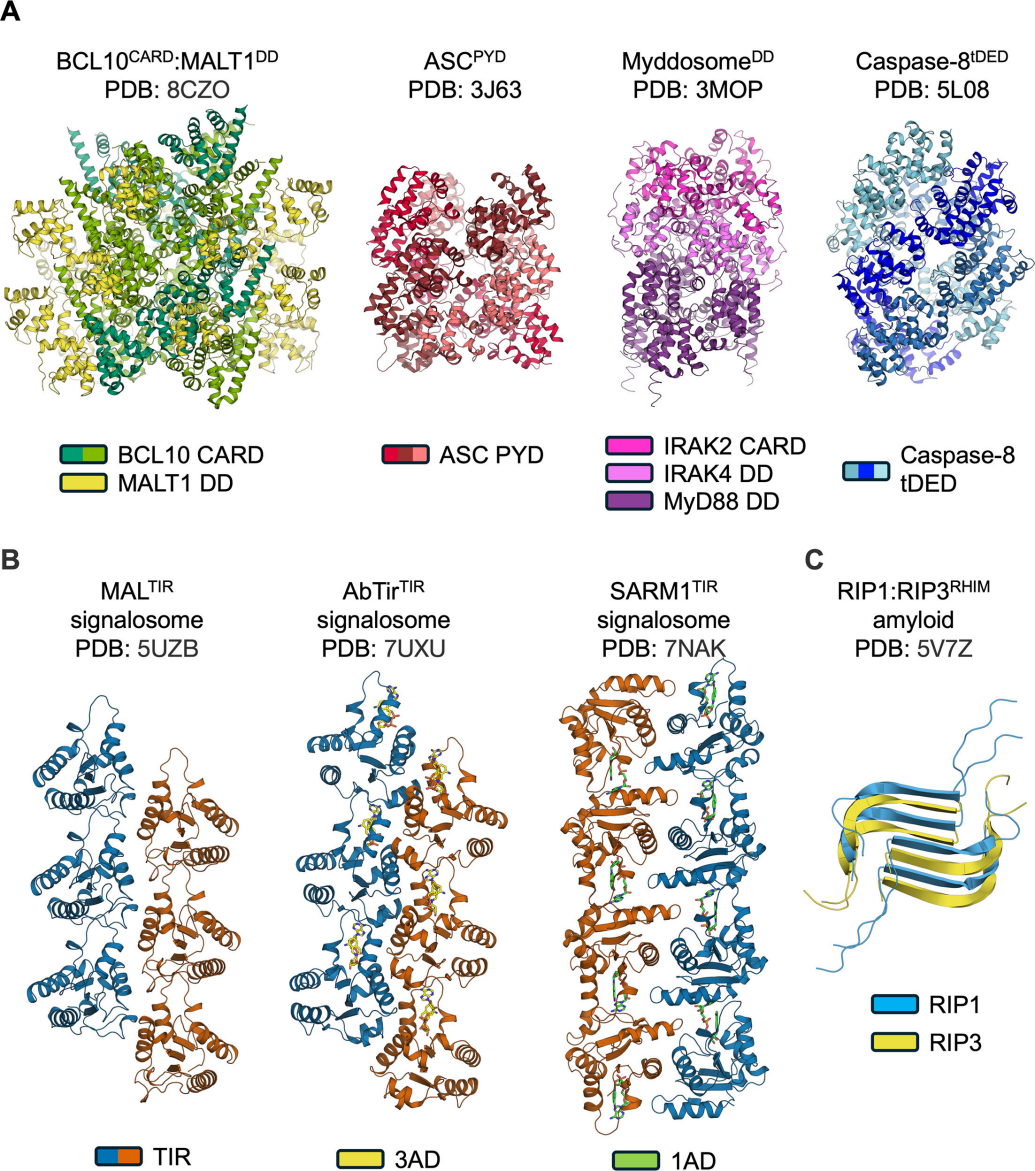
Structures of representative signalling assemblies consistent with the SCAF mechanism. (**A**) Structures of representative DF superfamily signalosomes: BCL10^CARD^ and MALT1^DD^ signalosome (PDB ID: 8CZO); ASC^PYD^ (PDB ID: 3J63) filament; DD Myddosome (PDB ID: 3MOP); and caspase-8^tDED^ (PDB ID: 5L08) filament. (**B**) Structures of representative TIR domain signalosomes: MAL^TIR^ (two-stranded parallel assembly; PDB ID: 5UZB), AbTir^TIR^ (two-stranded parallel assembly; PDB ID: 7UXU), and SARM1^TIR^ (two-stranded antiparallel assembly; PDB ID: 7NAK). 1AD (5-iodo-isoquinoline adenine dinucleotide) and 3AD (8-amino-isoquinoline dinucleotide) are NAD-mimetics. (**C**) Structure of the RIP1:RIP3 RHIM domain hetero-amyloid (PDB ID: 5V7Z). BCL10, B-cell lymphoma 10; DF, death fold; MALT1, mucosa-associated lymphoid tissue lymphoma translocation protein 1; NAD, nicotinamide adenine dinucleotide; RIP, receptor-interacting protein; SARM, sterile-alpha and Toll/interleukin-1 receptor motif; SCAF, signalling by cooperative assembly formation.

The DF superfamily includes the DDs, caspase recruitment domains (CARDs), pyrin domains (PYDs) and death effector domains (DEDs). Representative signalling assemblies formed by DF domains, TIR domains and RHIMs are summarized in Table 1 and Figure 2. For each domain superfamily, the corresponding assemblies feature structurally conserved arrangements. Whereas DF and TIR domain signalosomes are formed by folded domains, the RHIMs form an amyloid-like structure from motifs that are intrinsically disordered prior to assembly.

### SCAF mechanism

#### Co-operativity

The thermodynamic principle behind SCAF is positive co-operative assembly, where the binding of one protomer enhances the binding of subsequent protomers, by forming stabilizing interactions in different dimensions. The number of stabilizing interactions that can be made by a joining protomer depends on the number of protomers already in the oligomer. Co-operativity increases with the entropic cost of assembly. The loss of diffusive (intermolecular) entropy upon assembly sharpens the dependence of assembly on the concentration of protomers. Hence, larger assemblies tend to be more co-operative and, therefore, more sensitive. Conversely, the loss of conformational (intramolecular) entropy that can occur upon assembly sharpens the dependence of assembly on factors that influence the conformational ensemble of protomers, such as temperature, pH and cofactors.

Co-operativity becomes infinite when assemblies lack geometric constraints on their size, as for condensates and crystals. This creates a ‘phase boundary’, where small changes in concentration or conformation can drive assembly. SCAF appears to exploit this principle in two ways. First, SCAF involves transitions from monomers or small oligomers to massive assemblies whose sizes appear in some cases to be limited only by the number of protomers in the cell. Second, the transitions initiate with changes in the conformational ensembles of sensors, whereby PAMP/DAMP binding co-operatively releases autorepressed configurations of domains in the context of pre-assembled homooligomers that are poised to template adaptor assembly.

#### Nucleation barriers

While co-operativity sharpens the sensitivity of signalling responses, signal amplification to elicit a sufficient cellular response necessarily involves a different physical principle. The output of co-operativity is directly coupled to, and limited by, the energy of PAMP or DAMP binding. This is a problem because an appropriately sensitive response to infection requires recognition of PAMPs when they are at vastly substoichiometric levels relative to their receptors. Hence, tiny signals must be amplified to drive the requisite cellular responses, and amplification requires energy consumption [30–32]. Recent findings reveal that adaptor proteins typically serve this function, and the way they do so appears to have shaped the evolution of signalosomes’ peculiar filamentous structures [16,17].

Amplification permits signalling to be stoichiometrically uncoupled from the stimulus, so that numerous host proteins can be activated in response to even the smallest of genuine pathogen signals. SCAF facilitates this uncoupling through a structurally encoded phenomenon of phase transitions, dependent on supersaturation of a component, meaning that it exists in excess of its thermodynamic solubility limit in the cell. Just beyond a phase boundary (solubility limit), the degree of order required for an oligomer to grow is very large. The concentration of such oligomers, or ‘nuclei’, can be so low that a finite system such as a cell may contain fewer than one nucleus at any given moment. In this supersaturated regime, the assembly cannot proceed unless a nucleus spontaneously appears through a random local fluctuation in monomer concentration and structure. The greater the concentration of signalling proteins beyond the phase boundary (i.e. the more supersaturated the system), the greater the initial signal will be amplified by the resulting phase transition, once nucleation does occur. However, the concentration has to remain at a level where spontaneous assembly and signalling are very rare. Supersaturation serves as the energy source for the signalling response, replacing the more familiar energy sources such as ATP or electrochemical gradients [17].

The improbability of the nucleating fluctuation occurring at a given time and place is known as the nucleation barrier. The nucleation barrier falls as supersaturation increases because the critical size of nucleating oligomers decreases due to the ready availability of monomers for further polymerization, while the theoretical concentration of the nucleating oligomers increases. Cells cannot maintain highly supersaturating concentrations when nucleation is limited only by a density fluctuation [33]. For signalling proteins to achieve sufficient concentrations required for effective signalling responses, their co-operative activation must also involve an intramolecular (conformational) fluctuation, and the corresponding signalosome must exhibit ‘crystalline’ order. This order occurs in the form of DF and TIR domain filaments, and RHIM amyloids.

The nucleation barrier is ensured by the structural features of the signalosome. Deposition of molecules into the three-dimensional (3D) lattice of the corresponding polymers imposes a large entropic barrier, because the molecules need to be constrained to high local concentrations in the correct orientation, and a specific molecular conformation. Although the final signalosome structure is thermodynamically more stable, the nucleation barrier imposes a kinetic and, therefore, a probabilistic barrier. To limit aberrant signalling and ensure cell signalling only occurs to an appreciable extent within the context of the full signalosome, any individual bi-molecular interactions must be weak, for both receptors and adaptors. The binding of receptors to multivalent PAMPs stabilizes a weak interaction between the ligand and the receptor, increasing the probability of adaptor recruitment and, hence, reducing the entropic cost of forming a nucleating oligomer.

Ligand-bound receptors form dimeric or oligomeric complexes, with their signalling domains providing the nucleation template that overcomes the nucleation barrier, to allow supersaturated adaptors to drive signalosome assembly. Readers with experience in crystallography will be familiar with this phenomenon and the corresponding approach to increasing the size of crystals, using the seeding technique. Many readers will also be familiar with prions and amyloids, usually associated with pathological processes, that rely on the same mechanism. SCAF, therefore, allows a switch-like (digital) response, compatible with a binary decision that needs to be made when deciding between life and death. The corresponding signalosomes can, therefore, be considered analogue-to-digital converters. By amplifying weak signals, supersaturation ensures a decisive response to threats. The increased concentrations allow DF, TIR and RHIM monomers to assemble efficiently, enabling rapid and effective responses to pathogens and danger signals. The uniquely large nucleation barriers of disorder-to-order transitions explain why signalosomes in innate immunity feature ordered polymers so abundantly. With the nucleation barrier being such an important part of the mechanism, perhaps the ‘C’ (‘co-operative’) in SCAF could be replaced by ‘crystalline’.

The implications of the nucleation barrier from a cellular perspective were summarized by Halfmann and colleagues [22], who proposed the analogy with a spring-loaded mousetrap (Figure 1). The sensor protein represents the trigger of the mousetrap. The adaptors, which oligomerize in response to the sensor being triggered, represent the spring. The spring is loaded or tensed by the cell producing adaptor proteins in excess of their thermodynamic solubility limit in the cell. In this supersaturated state, the adaptor molecules are ready to oligomerize but are kept from doing so by the nucleation barrier. The binding of the sensor to the danger signal provides a template to mitigate the entropic cost of ordered assembly, lowering the nucleation barrier and triggering adaptor protein assembly. Once nucleated, adaptor protein assemblies continue to grow irrespective of the continued presence of the pathogen-related ligand, until a steady state is reached or a cellular response terminates the process. The assemblies bind and cluster together effector proteins to facilitate their trans-activation. The effector proteins then translate the signal into a cellular response, leading, for example, to programmed cell death. They represent the lethal hammer of the mousetrap.

### 2D/3D complexes, 1D filaments, amyloids and biomolecular condensates

Related processes of assembly involve one-dimensional (1D) polymerization into filaments, which is represented by sterile-alpha motif, dishevelled and axin, and Phox and Bem1 domains that are structurally related to each other [34,35]. This type of polymerization only requires one asymmetric interface (low valency) and lacks a nucleation barrier – assembly size increases with monomer concentration.

Another type of 3D assembly that has received a lot of recent attention is liquid–liquid phase separation (LLPS), the formation of biomolecular condensates [36]. This process involves multiple interactions, but those are not geometrically constrained (leading to more favourable configurational entropy). This process also features a nucleation barrier because of the entropic cost of bringing molecules together (density fluctuation); however, the barrier falls more sharply with concentration than for nucleation-limited SCAF featuring ordered assemblies and, therefore, is co-operative but not switch-like. The overlap and continuum between LLPS, hydrogel-like (e.g. FG-nucleoproteins in the nuclear pore complex [37]) and ordered 1D, two-dimensional (2D) and 3D assemblies (and their combinations) allows for a range of different behaviours that the cell can take advantage of to achieve the desired responses. These various assemblies feature different material states and dynamics (from liquids through hydrogels to solids), owing to the types and valencies of interactions. These interactions vary in affinity, stoichiometry, frequency of interacting elements and dynamics of the connecting regions (including the presence of intrinsically disordered regions) [38]. There are also opportunities for cross-talk between pathways that cells can take advantage of, including cross-seeding by different templates (again, a phenomenon well-known to crystallographers).

RHIMs are intrinsically disordered sequences of ~18–22 residues as monomers but assemble into an amyloid-like structure comprising stacks of RHIMs stabilized by forming β-sheets [39]. Amyloids have generally been associated with pathological processes such as degenerative and prion diseases and exhibit extreme stability; however, structurally analogous assemblies have subsequently been found to be involved in functional signalling and, therefore, termed ‘functional amyloids’ [40]. RHIM-like motifs comprise the largest group of functional amyloids and are found in diverse animals (exhibiting roles in necroptosis and innate immunity), fungi (exhibiting roles in heterokaryon incompatibility) and even prokaryotes. In necroptosis, there are, at least, three different pathways that lead to the permeabilization of the membrane by the effector MLKL (mixed-lineage kinase domain-like): through death receptor signalling, leading to the association of RHIMs from RIP1 and RIP3; through the activation of TLR3 or TLR4, leading to the association of RHIMs from TIR-domain-containing adaptor-inducing beta interferon (TRIF) and RIP3; and through sensing Z-form nucleic acids, leading to the association of RHIMs from Z-DNA-binding protein 1 (ZBP1) and RIP3 [39].

### Open-ended (filamentous) and closed (rotational symmetry) assemblies

When it comes to ordered 2D or 3D assemblies, they can feature an open-ended (filamentous) character or a closed complex featuring rotational symmetry. These alternatives provide another layer in the assembly continuum and are employed in cells to impose particular properties on the process, in terms of the nature of response and also in terms of evolutionary implications (newly acquired surface mutations more likely lead to symmetric self-association rather than open-ended assemblies) [41]. Notably, open-ended assemblies can be converted to closed ones by being stoichiometrically defined, as is the case for the DD Myddosome comprising 6, 4 and 4 DDs from MyD88, interleukin-1 associated kinase 4 (IRAK4) and IRAK2, respectively [4].

### Differences between SCAF-related assemblies and biomolecular condensates

**Figure 3:**
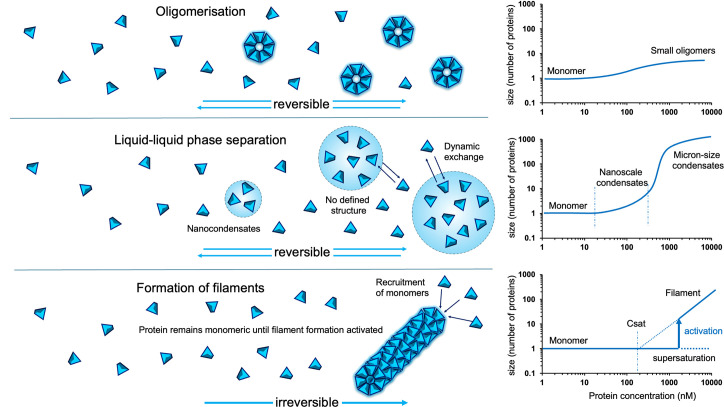
Comparison of equilibrium assembly formation in the case of small oligomers (top), LLPS (middle) and nucleated filaments (bottom). Contrary to the classic formation of oligomers, where particles assemble into small and well-defined structures, in LLPS, proteins can associate loosely into fluid condensates maintained by weak interactions. Both processes are reversible and do not present substantial nucleation barriers. In the case of filament formation, proteins are maintained in monomeric form even past a saturating concentration (C_sat_), thanks to a large nucleation barrier. When the system is activated, the proteins form filaments in an irreversible process, and monomers are recruited to the growing filaments. Past C_sat_, the system exists in two possible states: either in monomeric form in a supersaturated state, or polymeriszed if activated, with the polymer size increasing in a linear manner with protein concentration. LLPS, liquid–liquid phase separation.

In biomolecular condensates (Figure 3), proteins form a dense phase in small droplet-like structures. These condensates exist in equilibrium with a dilute phase of proteins that remain mostly monomeric. One of the most fascinating aspects of this process is that the assemblies are in a liquid phase, and the proteins can exchange freely between the dense and dilute phases. The condensates can form and dissolve rapidly; the formation of biomolecular condensates is often reactive to subtle changes in the cellular environment and can be triggered by changes in concentrations of proteins or nucleic acids [42,43].

In stark contrast with SCAF, there is no apparent structure within the condensates; proteins are associated loosely without stable interfaces. The droplets are rather stabilized by weak protein–protein or protein–nucleic acid interactions. While filament formation is mostly driven by homotypic interactions, and the assembly cores are formed from a single protein, condensates can encapsulate many different proteins, as was observed in stress granules, P granules and nucleoli [44–46].

Initially, transitions between the monomeric and phase-separated states in LLPS were observed as the formation of microscopic droplets [36,47]. So-called phase diagrams were established in systems reconstituted *in vitro,* and the critical concentrations for droplet formation were defined. More recently, it was shown that the transition is not as abrupt as originally thought and that nano-condensates are formed at the edges of the critical concentration [48,49].

Contrary to SCAF, proteins involved in LLPS do not exist at saturating concentrations in the cell (otherwise the droplets would form spontaneously); transitions are triggered by cellular changes, such as variations in local protein concentration. In LLPS, the sizes of assemblies are linked to the concentrations of components, whereas SCAF involves an all-or-nothing transition. Furthermore, LLPS is fully reversible and the droplets can dissolve rapidly, while polymer disassembly in SCAFs requires other mechanisms.

Nevertheless, there is increasing evidence for LLPS in immune signalling, including in T-cell and B-cell signalling and nucleic-acid sensing in the cGAS (cyclic GMP-AMP [cGAMP] synthase)-stimulator of interferon genes (STING) and retinoic acid-inducible gene I (RIG-I) pathways. T-cell activation is triggered when antigen binding to the TCR leads to the phosphorylation of the adaptor protein linker for activation of T cells (LAT), which, in turn, organizes in dense micro-clusters at the membrane that have liquid-like properties. Condensation of LAT increases the local density of adaptor and effector proteins and creates a hotspot for activation of downstream pathways [50]. Similar to LAT, the adaptor protein B-cell linker forms nanoclusters in B-cell signalling [51]. The mechanism is, however, different, as the condensates are formed as spherical objects in the cytoplasm and not as 2D rafts in the membrane.

The cGAS-STING pathway senses intracellular DNA from pathogens or DNA from damaged nuclei or mitochondria. cGAS has been observed to undergo condensation upon binding to double-stranded DNA (dsDNA) *in vitro* and in cells [52]. The formation of cGAS condensates enhances its activity by excluding an exonuclease and protecting the DNA from degradation. It is interesting that LLPS is also involved downstream of dsDNA recognition to modulate the immune response. When cGAS condensates produce too much cGAMP, condensation of STING occurs at the endoplasmic reticulum; these condensates sequester a specific protein kinase and prevent the overproduction of interferons [53]. Similarly, LLPS has been shown to drive signalling in the RIG-I pathway, which responds to single-stranded or dsRNA [54].

We are likely only scratching the surface in terms of the involvement of LLPS in innate immunity. For example, interferon regulatory factors 3 and 7, which drive the activation of type I interferons, also undergo LLPS [55]. Viruses can use LLPS as a strategy to block immune responses; condensates called inclusion bodies in host cells have been shown to trap p65, a subunit of the transcription factor nuclear factor-kappa B (NF-κB), to block its translocation and signalling in the nucleus, thus enhancing the replication of the viral genome [56].

LLPS and SCAF can work together. The formation of condensates can provide the initial trigger for filament formation. The concentration of proteins in condensates can be 10–100× more elevated than in the dispersed monomeric phase; this can push the system over the nucleation barrier. Recently, phase separation of NLR family pyrin domain containing 6 (NLRP6) with viral RNA *in vitro* and in cells was shown to trigger the formation of the NLRP6:ASC (apoptosis-associated speck-like protein containing a CARD) inflammasome [57]. Most characterized cases of LLPS have involved the detection of large condensates that can be easily detected in cells. It is probable that smaller, sub-micron condensates have not yet been detected due to the limitations of classic fluorescence microscopy.

### Implications of SCAF for cellular signalling

**Figure 4:**
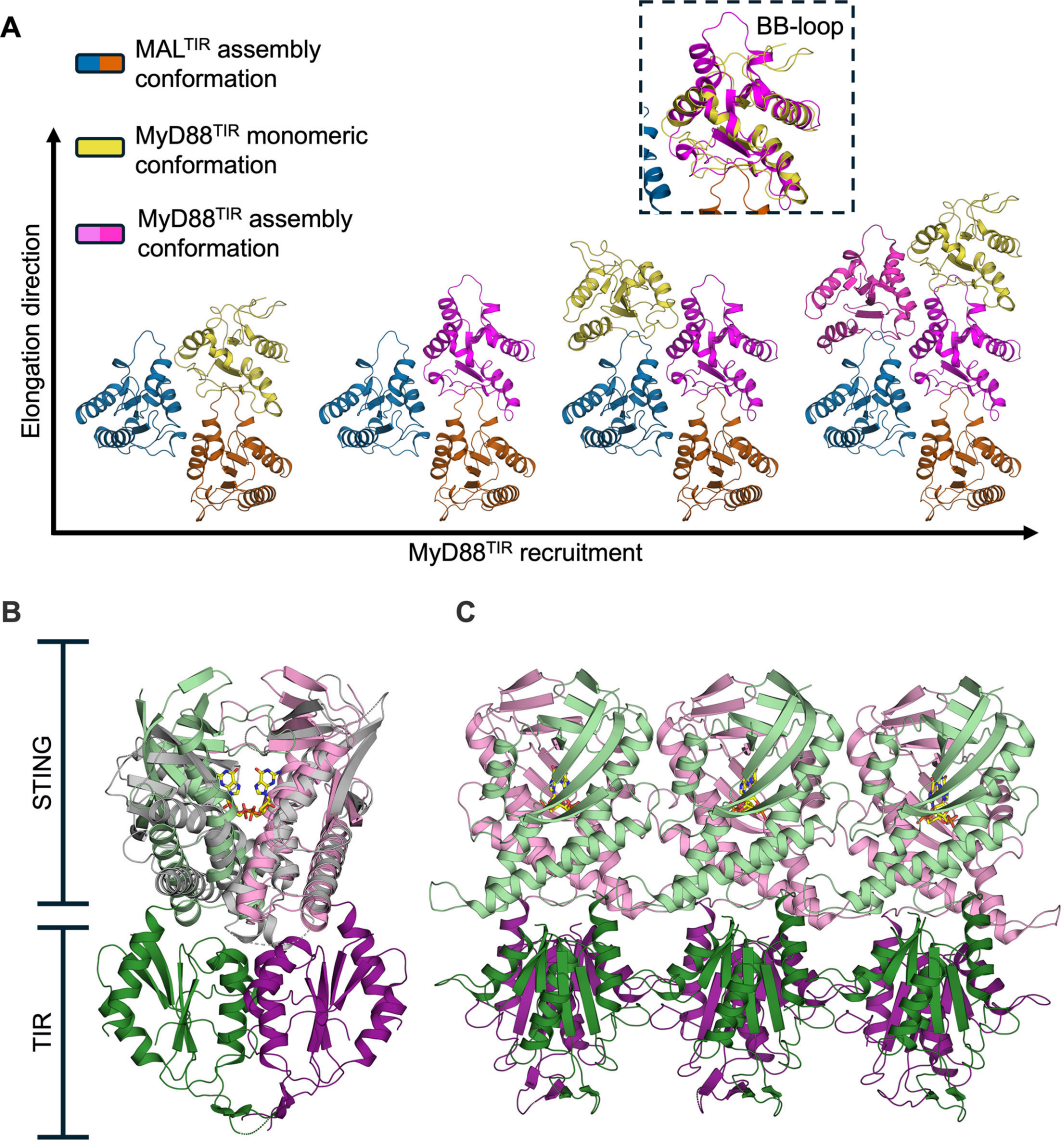
Conformational changes associated with signalosome assembly. Binding partners drive conformational changes required for co-operative assembly in many signalosomes. (**A**) A model of MyD88^TIR^ recruitment by MAL^TIR^ (PDB ID: 5UZB) and the resulting filament elongation. MAL^TIR^ serves a templating function, driving the initial recruitment of and conformational changes in monomeric MyD88^TIR^ (PDB ID: 2Z5V) required for the recruitment of new monomeric MyD88^TIR^ subunits. Interstrand interactions between the oligomeric MyD88^TIR^ (PDB ID: 7BER) and newly recruited MyD88^TIR^ induce conformational changes in the intrastrand interface (predominantly rearrangement of the BB-loop; shown in the inset) compatible with subsequent recruitment of additional MyD88^TIR^ subunits. (**B,C**) Cyclic di-GMP (yellow) produced by thebacterial CBASS (cyclic oligonucleotide-based antiphage signalling system) triggers activation of the bacterial NADase TIR-STING. Nucleotide binding by the STING sensor domain of inactive TIR-STING dimers (grey; PDB ID: 6WT5) induces tight association of the STING domains (light green/light pink) (**B**). Conformational changes in the STING domains subsequently induce self-association of the effector TIR domains (dark green/dark pink) and the formation of catalytically active filaments (PDB ID: 7UN8) (**C**). MAL, MyD88 adaptor-like; STING, stimulator of interferon genes; TIR, Toll/interleukin-1 receptor.

Classical signalling pathways, such as those involving G protein-coupled receptors) and receptor tyrosine kinases that involve multiple rounds of catalytic actions (‘chain reaction’), allow a graded signalling response and the regulation of the lifetime of signalling through counter-enzyme deactivation (e.g. phosphatases in the case of phosphorylation). The SCAF mechanism instead brings a set of specific features, including proximity-based enzyme activation, threshold behaviour, large signal amplification, reduction in biological noise, and temporal and spatial control. Co-operativity results in non-linear, sigmoidal dose responses (described by the Hill coefficient). Co-operativity depends on simultaneous interactions with multiple binding partners and/or conformational (often allosteric) changes (Figure 4). The threshold behaviour ensures a maximal sensitivity of response, while ignoring stimuli that do not reach the threshold (i.e. ‘biological noise’). The portion of the signalling pathway involving SCAF mechanisms typically does not involve second messengers, but in some instances, TIR domains have enzymatic activity and can themselves generate second messenger molecules [58]. The mechanism also provides resilience against inhibitors from pathogens, considering that the fast activation allows little time for inhibitors to act [59].

The binary signalling response is most clearly seen with the inflammasome, where there is potential for triggering even a single inflammasome receptor complex in turn leading to polymerization of the majority of the ASC adaptor in the cell, resulting in the recruitment and activation of caspase-1 and pyroptotic cell death. However, in the case of SCAF processes with multiple smaller complexes (e.g. TLRs), the response could be graded at the cellular level. Although the signalling outcome is binary for an individual receptor complex, the number of responding receptors and, hence, signalosomes formed can determine the overall signal strength.

### Disassembly of signalosomes

Processes such as autophagy may be adequate to eliminate the signalosomes after signalling has run its course. Autophagy appears to be a process used in a number of different pathways (e.g. B-cell lymphoma 10 [BCL10]), mitochondrial antiviral signalling [MAVS] protein, ASC and MyD88 [60–62]).

In cases where the cell dies, there may be no immediate need for the signalosome to be disassembled after it has done its job; the reversibility of assembly may, therefore, not be required. In fact, cell death may lead to the release of signalosomes. These extracellular assemblies may be long-lived, as suggested for ASC complexes that can be detected in serum and cerebrospinal fluid of patients with inflammatory diseases, which can be harnessed as a diagnostic marker [63]. The physiological implications for extracellular signalosomes are incompletely understood but may reach beyond their initial signalling functions, e.g. as seeds for pathological protein aggregation [64].

RHIM amyloids involved in necroptosis can be disassembled by the heat-shock protein HSPA8, acting as an amyloidase preventing necroptotic cell death [65]. The discovery of this enzyme adds to the understanding of a key regulatory aspect of signalling by functional amyloids, for which reversibility must be an essential feature distinguishing them from pathological amyloids.

### Cellular evidence for SCAF

#### Signalosome sizes in cells

The initial supporting evidence for SCAF operating in cells relates to the observation of punctate morphologies of signalosomes in cells (e.g. Fas-FADD [66]; RLRs [67–69]; inflammasomes [6,70–74]; TLR pathway components [75–80]; and cGAS-STING [81,82]). In agreement, in the case of the CARD–BCL10–MALT1 (CBM) signalosome, only high molecular-weight fractions of signalling proteins showed activity [83]. The filamentous nature of some of these assemblies may not always be apparent due to the dense packing of filaments. The best example of this is the observation of the 1-µm spherical ‘specks’ of the inflammasome adapter molecule ASC. Within minutes of inflammasome sensor protein activation, the bulk of the ASC in the cell redistributes from a diffuse localization to a dense cytosolic speck structure [84]. This speck is built by helical filaments of the ASC N-terminal PYD [6]. ASC also contains a C-terminal CARD; apart from recruiting caspase-1, it can form ASC CARD–CARD interactions that mediate filament branching, as well as condensation into the tight speck structure. The filamentous nature of the ASC assembly was well demonstrated using intracellular expression of a nanobody that targeted the CARD and prevented speck condensation [85].

The situation in TLR pathways is more complex, presumably because of the lower stability of TIR-domain assemblies and the stoichiometric nature of the DD Myddosome. Latty et al. [86] used single-molecule imaging to follow TLR4 and MyD88 during signalling in macrophages. Halo-tagged TLR4 was found to exist in an equilibrium of heterodimeric and hetero-tetrameric TLR4:MD2 (myeloid differentiation factor 2) complexes on the cell surface, with the latter species being enriched upon agonist (LPS, lipopolysaccharide) treatment; no higher-order species were detected. Total internal reflection fluorescence microscopy was used to visualize fluorescently labelled MyD88 recruited to the cell membrane, revealing complexes of the size of six molecules (and under some conditions, ‘super-Myddosomes’ containing 12 molecules), which rapidly moved away from the membrane. Correlating these results with the activation of the transcription factor NF-κB, the authors proposed that agonists stabilize the activated form of the receptor dimer, promoting the formation of Myddosomes containing only 6–12 molecules, which are short-lived at the cell surface.

Deliz-Aguirre et al. [87] visualized dynamics of MyD88 in living cells. They found the complex to be initially reversible, but with more than four molecules, it became more stable and recruited IRAK1 and 4, with IRAK4 controlling the size of the complex. The authors concluded that MyD88 cluster size determines the physical threshold for downstream signalling.

A recent extensive study of MyD88 signalling in macrophages, using microscopy and proteomics, added further details to the complex set of events taking place during the signalling response [62]. Upon receptor activation, MyD88 molecules formed only small clusters (around six molecules) at the membrane and moved away from the membrane (dissociating from the receptors) within 30 min, forming larger clusters in the cytoplasm. These large complexes, some of which presented as barrel-like structures, persisted until around 12 h, when they were cleared by autophagy. The large barrel-like complexes contained a range of proteins categorized into Myddosome core proteins, signalling proteins, autophagy-related proteins and interferon-stimulated gene products. IRAK4 was found to be the key component controlling the dynamics of these large clusters. The emergence of large clusters that harbour signalling and regulatory proteins does not exclude the possibility that the SCAF-related portion of signalling takes place at the membrane, largely regulated by TIR domain associations, which, then, terminates after the movement from the membrane and allows the large cytoplasmic clusters to orchestrate later signalling events. The study highlights the differences in stability of TIR-domain and DF-superfamily signalosomes, consistent with the observations using an engineered single-component signalosome (see below) [88].

In the case of both inflammasome specks and MyD88 complexes that reach up to 1 µm diameter, it should not be assumed that such massive complexes are essential to the signalling process. The critical signalling events may have happened at a much earlier stage on polymers of limited size, with the continued assembly, an inevitable consequence of supersaturation and thermodynamic considerations.

### Are signalosomes defined *in vitro* representative of complexes present in cells?

Mutagenesis coupled with cellular assays showed, for virtually all signalling assemblies discussed in this article, that the disruption of interactions observed in reconstituted assemblies studied structurally leads to the disruption of signalling (see specific references to particular structures). Constraints imposed by the cellular environment and specific localization of signalling may affect the signalosome size in cells. The emerging technique of cryogenic electron tomography (cryo-ET) promises the visualization of signalling assemblies directly in cells vitrified in an active signalling state, at near-atomic resolution [89]. ASC puncta have recently been imaged in immortalized primary mouse bone marrow-derived macrophages by correlative fluorescence light microscopy and *in situ* cryo-ET, which revealed a network of branched filaments with a tubular core of similar dimensions to filaments formed by purified ASC PYDs [90].

### Co-operativity, signal thresholds and biological noise reduction

The role of co-operativity, in terms of both protomer density and allostery, is evidenced by the extraordinary sensitivity of pathogen-responsive pathways to relatively small structural or concentration differences in stimuli. For example, cGAS in THP-1 cells responded strongly to 45 bp DNA at 10 nM concentration, but not at 1 nM concentration, nor to 25 bp DNA even at 100 nM concentration [91].

Co-operativity helps signalling pathways to avoid activating in response to noise. Microfluidic single-cell experiments showed that digital signalling by the NF-κB pathways (a transient strong stimulus) led to rapid and uniform output dynamics in a heterogenous cell population, suggesting efficient reduction in biological noise [92,93].

### Switch-like activation and amplification through supersaturation

The extreme signal amplification that is required for innate immunity is evidenced by the switch-like responses of innate immune signalling pathways to small stimuli, which have been observed in a range of cell-decision pathways including inflammasome, CBM signalosome and NF-κB signalling [16,62,92,94–99]. It has been estimated that the RIG-I signalling pathway activates with fewer than 20 copies of viral RNA in the cell [100]. A single bacterium contains a 1000-fold more LPS than required to activate a macrophage through TLR4 signalling [101]. Interestingly, co-activation of NF-κB signalling with different TLR inputs (TLR2 and TLR4) showed that individual cells retained ligand-specific responses, rather than a ‘mixed’ response, prompting the cellular decision to be labelled ‘non-integrative processing’ [102].

Signal amplification using the SCAF mechanism requires that at least one of the adaptors is present in a supersaturated state in cells. It was initially shown that the adaptor proteins MAVS and ASC can supersaturate in cells [103]. Subsequent work revealed that both ASC and another adaptor, BCL10 of the CBM signalosome, are supersaturated in their endogenous prestimulated states [16,17]. In both cases, supersaturation results from large sequence-encoded nucleation barriers that are conserved across metazoa [16,17]. This property is almost certainly not limited to these two proteins. A systematic analysis of phase behaviour by all 109 human DF domains using distributed amphifluoric fluorescence resonance energy transfer identified 17 that can supersaturate in cells. Nucleating interactions by the different DF domains proved to be highly specific and generally constrained to members of the same pathway, consistent with strong selective pressure to minimize spurious activation. Intriguingly, adaptor proteins were especially supersaturable, with most pathogen-responsive signalosomes containing one such adaptor. A notable twist on this paradigm was the Myddosome. Consistent with the role of co-operativity, this signalosome assembles only above threshold concentrations of MyD88 [62,104]. However, the DD of this adaptor is not supersaturable [17], while the upstream TIR domain-containing adaptor, TRIF , is [105,106], suggesting that whereas DF domain filament formation amplifies signalling for most pathogen-responsive pathways, TIR domains fill this need for the Myddosome.

### Signalosome stability and avidity

To define the unifying design principles of SCAF signalosomes, Lichtenstein et al. used a bottom-up approach, building a simplified signalling system they termed CHARMS (chimeric higher-order assemblies for receptor-mediated signalling), a single-component signalosome composed of MyD88 fused to the tumour necrosis factor receptor-associated factor 6 (TRAF6) interaction motif from IRAK1, removing the requirement for IRAK proteins in the Myddosome [88]. This construct mimicked the native system in terms of NF-κB activation and downstream transcriptional responses. The authors found that they could replace MyD88 DD with a bacterial DD or a synthetic filament-forming domain, suggesting that the stability of the interaction, rather than specific structural properties, is key to the signalosome function. The MyD88 TIR domain alone could not function without the DD, suggesting that its assembly does not provide the required stability. The inclusion of several TRAF6 interaction motifs increased the amplitude of signalling outputs. These results highlight two key parameters required for functionality: the signalosome kinetic stability and the local density of effector recruitment, both of which can be used to control the properties of signal transduction.

### Beyond the digital response

While SCAF responses are digital at the signalosome or cellular levels, ‘continuous’ or graded responses that scale with the amount of stimulus can be achieved at the multicellular level or even within single cells. At the multicellular level, this is achieved by heterogeneous expression of signalosome components, so that individual cells exhibit different sensitivities to stimuli and/or different levels of output. Death receptor-mediated activation of apoptosis typically exhibits such heterogeneity, wherein a fraction of cells even in clonal populations fail to respond [107]. At the individual cell level, the number of responding receptors and, hence, signalosomes formed could determine the overall signal strength, despite the signalling outcome being binary for an individual receptor complex. Following MyD88 complexes using live cell imaging, it was found that the clustering of Myddosomes controls digital versus analogue signalling [108]. In the context of TLR stimulation, the number of Myddosomes per cell and rate of their formation within minutes post-stimulation have been implicated as determinants of both graded TLR-mediated intracellular signalling and target gene expression [62,86]. Therefore, despite the switch-like nature of SCAF, graded signalling properties can be realized.

### Safety mechanisms and relevance to pathology

#### Risk mitigation of inappropriate activation

While the SCAF mechanism features properties that are beneficial for innate immunity and cell-death responses, it comes with an inherent risk to the cell that it triggers at an inappropriate time. Such risks are not limited to inflammatory disease; excess proliferation can lead to cancer or inappropriate cell death could cause tissue damage. Cells mitigate this risk through a variety of mechanisms.

One safety mechanism is to only arm the system when danger is imminent, through priming by way of transcriptional and post-transcriptional mechanisms and post-translational modifications (e.g. in the case of the NLRP3 inflammasome [109]). Conversely, negative feedback regulation can modulate the pathways after activation, such as through the disassembly of complexes as discussed earlier.

An additional safety mechanism involves the sensors often being present in an autoinhibited form and converting to the active form through conformational changes upon binding of the danger signal. These danger signals can be either multivalent (i.e. nucleic acids interacting with TLRs [110], RLRs [111,112] and AIM2 [113]), or monovalent (e.g. nicotinamide mononucleotide interacting with sterile-alpha and Toll/interleukin-1 receptor motif-containing 1 [SARM1] [114], or pathogen proteins interacting with NLRs [115–117]). The sensors can oligomerize themselves (e.g. NLRs; reviewed in [115,116,118]); or are pre-assembled in an oligomeric form, but their signalling components are held in an inactive conformation (e.g. SARM1; reviewed in [119]). While the 2D membrane environment provides energetic advantages for protein−protein interactions, localization of the pathway to a membrane or particular part of the cell further constrains and fine-tunes the outputs of the signalling event [18].

Mutations that disrupt safety-related features such as autoinhibition and lead to constitutive activation represent a longer-term risk; they can cause pathological outcomes such as inflammatory disease. Another risk is the probabilistic nature of the kinetic barrier, which can manifest in age-related problems; the cells harbouring supersaturated adaptors are at the risk of eventual spontaneous activation of inflammation. Cells employ a number of regulatory proteins that can modulate signalosome assembly and tune the nucleation barrier (but similar approaches have also been adopted by pathogens for their own benefit; reviewed in [120]). For example, the filamentous signalosomes formed by caspase-1 CARDs can be capped by the CARD-only protein INCA (inhibitor of CARD) and those formed by caspase-8 by the DED-containing protein MC159 (protein from the Molluscum contagiosum virus) [3,121]. By contrast, the CARD-only protein ICEBERG (interleukin-1 converting enzyme/caspase-1–BERG) and the DED-containing protein cFLIP (cellular caspase-8 [also called FLICE] inhibitory protein) incorporate into signalling filaments and presumably interfere with proximity-based activation of caspases [3,121]. For down-modulation of inflammatory signalling, a splicing variant of MyD88 (MyD88S), expressed only after continuous stimulation, was proposed to interfere with signalosome assembly through a capping effect [105]. Pathogens have also evolved mechanisms to interfere with or regulate SCAF-mediated signalling. The A46 protein from the vaccinia virus targets and disassembles the MAL (MyD88 adaptor-like) and MyD88 signalosome [122]. Viruses and bacteria have evolved to evade necroptosis, either by expressing RHIM-containing proteins (e.g. herpes simplex virus-1 protein ICP6 [infected cell protein 6] that interacts with host proteins ZBP1 and RIP3), or by cleaving the host RHIM proteins (e.g. the enteropathogenic *Escherichia coli* protease EspL) [39].

### Relevance to pathology beyond inflammatory diseases: the case of MyD88

MyD88 is the adaptor downstream of most TLRs and a central node for immunomodulation [123]. It is over-expressed in many cancers and plays a role in their initiation and progression (reviewed in [124]). In colorectal and ovarian cancers, for example, MyD88 expression levels correlate with poor prognosis and reduced survival. Over-activation of MyD88 is a recognized driver of carcinogenesis in B-cell lymphomas. MyD88 mutations are found in large proportions of diffuse large B-cell lymphomas [125]. The oncogenic MyD88 mutations are gain-of-function mutations that lead to increased IRAK1 binding and downstream signalling.

Structurally, most mutations are found in the BB-loop of the TIR domain, a region involved in filament formation [7,8,126]. At the molecular level, MyD88 mutants were found to have an increased aggregation propensity. This is particularly true for the most common MyD88 mutation L252P (also referred to as L265P). This mutation is found in >95% of Waldenström’s macroglobulinemia cases, a rare B-cell non-Hodgkin lymphoma; it is used in differential diagnosis [127]. Structurally, the corresponding residue has been proposed to allosterically modulate other regions of the protein; molecular simulations suggest the mutation increases the stability of oligomers [128]. In a reconstituted system, the L252P mutant was found to form smaller assemblies than wildtype MyD88 but could self-associate at less than 2% of the concentration of the wildtype protein [106].

The link between aberrant clustering and over-activation of inflammatory pathways offers new therapeutic perspectives, including in cancer therapy. Beside B-cell lymphomas, where MyD88 activation is a direct promoter of carcinogenesis, dysregulation of MyD88 can have direct effects on tumour progression or indirect effects on the immune microenvironment of the tumour [124]. Therefore, the development of inhibitors targeting the self-assembly of MyD88 is an area of growing interest. Initial proof-of-principle was established with peptidomimetics mimicking the TIR-domain BB-loop discussed above, demonstrating that the disruption of MyD88 interactions leads to a reduction in immune responses [129,130]. Treatment with the peptidomimetic showed beneficial effects in cell models of several cancers, including in cells expressing the L252P mutant [131–134]. Similar effects were also reported for the small molecule lasalocid A, which targets MyD88 L252P for degradation [135]. Co-treatment with compounds targeting proteins downstream in the signalling pathway, such as Bruton’s tyrosine kinase and BCL2, showed synergistic effects. Overall, these results make MyD88 inhibition an enticing target for oncology. Multiple groups are rationally designing small molecules to prevent MyD88 interactions (e.g. [136–140]). None of the inhibitors have yet been tested in clinical trials though, and their therapeutic potential remains to be validated. Successful results could suggest that other signalosomes may be suitable targets.

### Open questions and future directions

One interesting aspect of the evolution of SCAF signalosomes is that their structures evolve according to both their kinetic and thermodynamic (equilibrium) properties. It is clear that specific modules are repeatedly used across pathways and organisms in innate immunity pathways (e.g. TIR domains in innate immunity in plants and antiphage defence in bacteria [58] and repeat domains in sensor proteins [141–143]). However, it is often not clear if pathways have a common origin (i.e. if they are related by divergent evolution) despite sharing homologous domains. Homologous domains may frequently be recruited by horizontal gene transfer, and the pathways may, therefore, evolve by convergent evolution [59,144].

Another question is whether signalosomes have context-dependent components that are cell-type or organelle-specific. Beyond being restricted to individual cells, there is some evidence for signal transduction between cells [145,146]; how common this is, remains an open question.

There may be pathways beyond innate-immunity and cell-death pathways highlighted in our article that incorporate features of the SCAF mechanism, such as the ER unfolded protein stress response and nucleic acid-associated filaments in homologous recombination and DNA replication [147,148]. Recent progress in identifying antibacteriophage signalling pathways in bacteria [149,150] has uncovered numerous proteins containing SCAF-related domains, including TIR and DF domains [151–154]. Indeed, bacterial TIR domains have been shown to form filamentous assemblies (Figures 2B and 4BC,). These observations support the concept of ‘ancestral immunity’ – conservation of domains and proteins involved in this process from prokaryotic to eukaryotic species [155] – and suggest that SCAF is an ancient mechanism that operates in prokaryotes and eukaryotes. The domains in bacterial proteins represent an enormous resource of sequence diversity that will, no doubt, help understand the evolution and mechanism of SCAF.

The SCAF concepts discussed in the present study may find practical application in synthetic biology, nanotechnology or the development of therapeutics, such as drugs aimed at modifying nucleation barriers. In conclusion, while the SCAF signalosomes are structurally diverse, they share key mechanistic features that are becoming increasingly understood.

## Abbreviations

AIM2: absent in melanoma 2
ASC: apoptosis-associated speck-like protein containing a CARD
BCL2/10: B-cell lymphoma 2/10
BCR: B-cell receptor
CARD: caspase recruitment domain
CBM: CARD–BCL10–MALT1
DAMP: damage-associated molecular pattern
DED: death effector domain
DF: death fold
DISC: death-inducing signalling complex
FADD: Fas-associated death domain protein
GPCR: G protein-coupled receptor
IL-1R: interleukin-1 receptor
INCA: inhibitor of CARD
IRAK: interleukin-1 receptor-associated kinase-like
LAT: linker for activation of T cells
LLPS: liquid–liquid phase separation
LPS: lipopolysaccharide
MAL: MyD88 adaptor-like (also called TIRAP, TIR domain-containing adaptor protein)
MALT1: mucosa-associated lymphoid tissue lymphoma translocation protein 1
MAVS: mitochondrial antiviral signalling protein
MD2: myeloid differentiation factor 2
MyD88: myeloid differentiation primary response 88
NAD: nicotinamide adenine dinucleotide
NF-κB: nuclear factor-kappa B
NLR: nucleotide-binding oligomerization domain-like receptor
NLRP3: NLR family pyrin domain containing 3
PAMP: pathogen-associated molecular pattern
PIDD: p53-induced protein with a death domain
PYD: pyrin domain
RHIM: RIP homotypic interaction motif
RIG-I: retinoic acid-inducible gene 1
RIP(K): receptor-interacting protein (serine/threonine-protein kinase)
RLR: RIG-I-like receptor
RTK: receptor tyrosine kinase
SARM1: sterile-alpha and Toll/interleukin-1 receptor motif-containing 1
SCAF: signalling by co-operative assembly formation
SMOC: supramolecular organizing center
STING: stimulator of interferon genes
TCR: T-cell receptor
TIR domain: Toll/interleukin-1 receptor domain
TLR: Toll-like receptor
TRAF6: tumor necrosis factor receptor-associated factor 6
TRAM: TRIF-related adaptor molecule (also called TICAM-2, TIR domain-containing adaptor molecule 2)
TRIF: TIR-domain-containing adaptor-inducing beta interferon (also called TICAM-1)
ZBP1: Z-DNA-binding protein 1
cFLIP: cellular caspase-8 [also called FLICE] inhibitory protein
cGAS: cyclic GMP-AMP synthase
